# PKM2-driven metabolic reprogramming in digestive system tumors: mechanisms, therapeutic advances, and clinical challenges

**DOI:** 10.3389/fimmu.2025.1634786

**Published:** 2025-08-06

**Authors:** Xinyao Huang, Jianjun He, Haonan Sun, Yi Wu, Renjun Gu, Ziyun Li

**Affiliations:** ^1^ The First Clinical Medical College, Nanjing University of Chinese Medicine, Nanjing, China; ^2^ Department of Radiation Oncology, Jinling Hospital, Affiliated Hospital of Medical School, Nanjing University, Nanjing, China; ^3^ Department of Infectious Diseases and Hepatology, Jinling Hospital, Affiliated Hospital of Medical School, Nanjing University, Nanjing, China; ^4^ School of Chinese Medicine, Nanjing University of Chinese Medicine, Nanjing, China; ^5^ Department of Gastroenterology and Hepatology, Jinling Hospital, Affiliated Hospital of Medical School, Nanjing University, Nanjing, China; ^6^ School of Acupuncture-Moxibustion and Tuina, School of Health Preservation and Rehabilitation, Nanjing University of Chinese Medicine, Nanjing, China

**Keywords:** PKM2, metabolic reprogramming, digestive system neoplasm, glycolysis, Warburg effect, oncotherapy

## Abstract

Metabolic reprogramming is a central driving force in the malignant progression of digestive system tumors. It facilitates tumor proliferation, metastasis, and therapeutic resistance through aerobic glycolysis, disordered lipid metabolism, and altered amino acid metabolism. Pyruvate kinase M2 (PKM2) functions as a key regulator of tumor metabolism, promoting aerobic glycolysis and suppressing mitochondrial respiration via conformational changes and nuclear translocation. These processes are orchestrated by hypoxia-inducible factors and oncogenic signaling, ensuring a sustained energy supply and biosynthetic precursors for tumor growth. Additionally, PKM2 modulates lipid biosynthesis and amino acid metabolism by participating in epigenetic regulation and the organization of metabolic enzyme complexes. These functions contribute to tumor adaptation within the microenvironment and promote immune evasion. In digestive system tumors, the regulatory network of PKM2 demonstrates tissue specificity, mediated by non-coding RNAs, post-translational modifications, and crosstalk between metabolic and signaling pathways, collectively sustaining metabolic plasticity. Therapeutic strategies targeting PKM2 primarily aim to reverse the Warburg effect or inhibit compensatory metabolic pathways; however, their clinical translation remains challenging. The dual regulatory role of PKM2 may perturb immunometabolic homeostasis; the fluctuating nutrient landscape of the tumor microenvironment can drive adaptive resistance; and some inhibitors exhibit limited specificity or unacceptable toxicity. This review summarizes the molecular mechanisms through which PKM2 drives metabolic reprogramming in digestive system tumors, as well as the current therapeutic advances and clinical barriers.

## 1 Introduction

Metabolic reprogramming is a hallmark of cancer and plays a central role in tumor initiation, progression, and metastasis by coordinating intra- and extracellular signals to promote malignant phenotypes (1). To sustain rapid proliferation, tumor cells reshape their metabolic pathways to meet energy and biosynthetic demands and to adapt to microenvironmental stress (1). In digestive system cancers-including hepatocellular carcinoma (HCC), gastric cancer (GC), and colorectal cancer (CRC)-distinct metabolic alterations are observed, such as enhanced aerobic glycolysis, dysregulated glutamine utilization, and abnormal lipid synthesis. These changes not only support tumor growth but also promote invasion and metastasis (1, 2). Additionally, the accumulation of metabolites like lactate contributes to an immunosuppressive microenvironment, impairing immune cell function and facilitating immune escape and therapeutic resistance (3).

Pyruvate kinase M2 (PKM2), a rate-limiting enzyme in glycolysis, is a key driver of metabolic reprogramming in cancer by promoting the Warburg effect (4). It is frequently overexpressed in tumor tissues and correlates with poor prognosis (4). Even in the presence of sufficient oxygen, PKM2 favors aerobic glycolysis over mitochondrial respiration, leading to increased lactate production and supplying energy and biosynthetic substrates to support tumor growth (5). In addition to its enzymatic role, PKM2 regulates tumor proliferation, metastasis, and apoptosis through non-metabolic functions, including its activity as a protein kinase (6).

Beyond its metabolic role in tumor progression, PKM2 also participates in remodeling the tumor immune microenvironment. In digestive system malignancies, SUMOylated PKM2 can be secreted via exosomes and internalized by immune cells, where it activates STAT3 signaling and reprograms their metabolic and functional states (7). In pancreatic ductal adenocarcinoma, tumor-associated macrophage–derived TGF-β1 induces PKM2 nuclear translocation, which activates STAT1 signaling and modulates immune checkpoint pathways (8). These findings underscore the immunomodulatory potential of PKM2 within gastrointestinal tumors and highlight its multifaceted role beyond metabolism.

**Figure 1:**
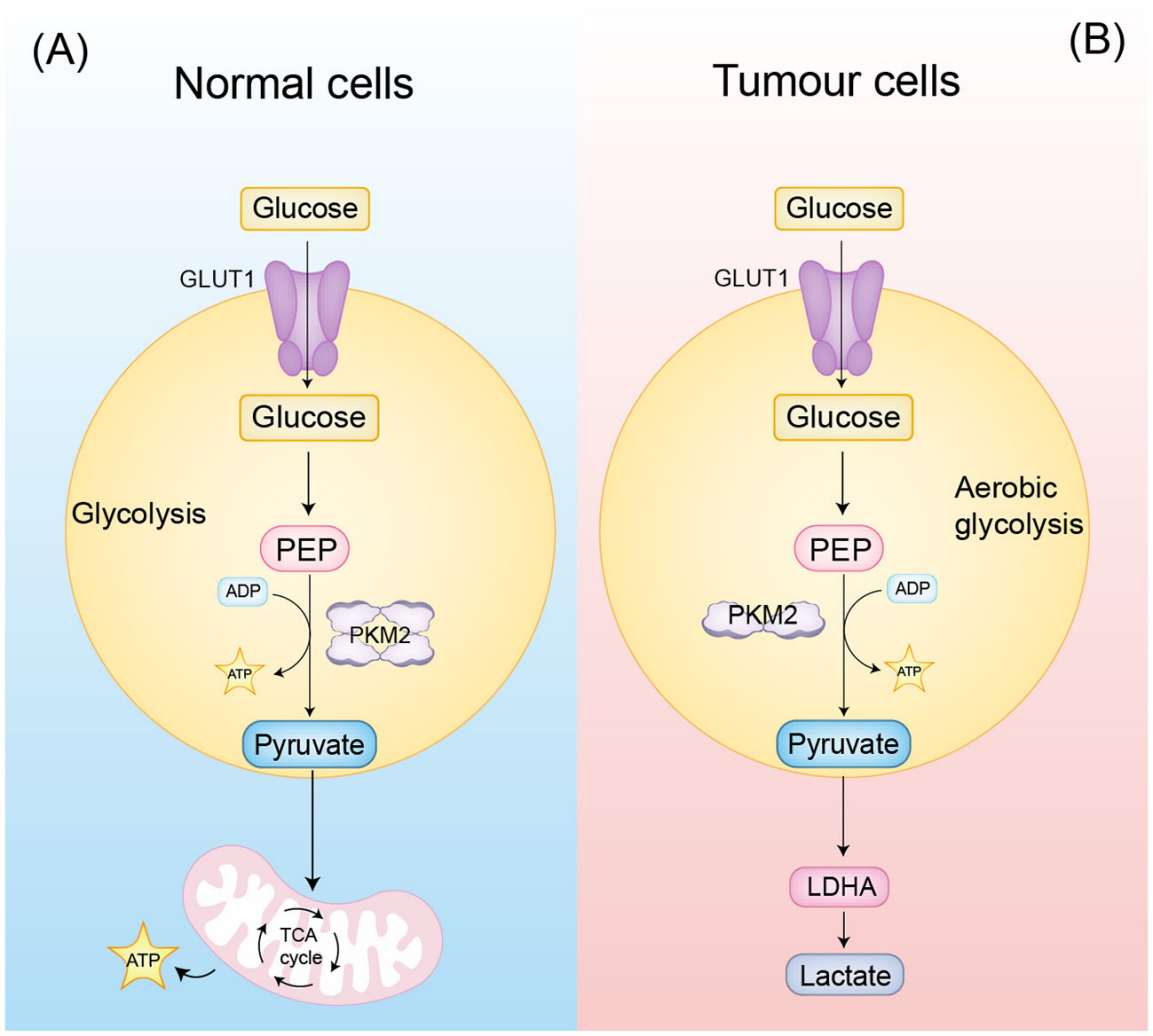
PKM2 classical function **(A)** In normal cells, PKM2 tetramers are involved in glycolysis. **(B)** In tumor cells, PKM2 dimers are involved in aerobic glycolysis, producing more lactate and further remodeling the tumor microenvironment.

Building upon this dual role in metabolism and immune regulation, recent studies have further revealed that PKM2 is intricately involved in the development and therapy resistance of digestive system tumors. It exerts its effects by modulating structural conformation, engaging in epigenetic regulation, and interacting with diverse metabolic and signaling networks (5). Based on these insights, this review summarizes the molecular mechanisms by which PKM2 drives metabolic reprogramming, outlines tumor-specific regulatory networks across various digestive system malignancies, including hepatocellular carcinoma (HCC), gastric cancer (GC), and others, and discusses current therapeutic strategies targeting PKM2 along with challenges in clinical translation. Together, these perspectives provide a foundation for future research and the development of precision treatment approaches (**Figure 1**).

## 2 The role of PKM2 multifunctional hub

### 2.1 Metabolic regulatory functions of PKM2

PKM2 primarily facilitates tumor-specific metabolic reprogramming by forming low-activity dimers that favor the final step of glycolysis, leading to pyruvate accumulation while suppressing mitochondrial respiration, thereby reinforcing the Warburg effect and promoting rapid cancer cell proliferation (9). It maintains metabolic homeostasis via several mechanisms: promoting Mitofusin 1/2 (MFN1/2)-mediated mitochondrial fusion to stabilize membrane potential (10), modulating the Nicotinamide Adenine Dinucleotide Phosphate (NADPH)/Glutathione (GSH) axis to maintain redox balance (11), and supporting Phosphoglycerate Dehydrogenase (PHGDH)-dependent serine synthesis to fulfill biosynthetic demands (12). PKM2 also contributes to therapy resistance by upregulating Death-Ligand 1 (PD-L1) expression through Signal Transducer and Activator of Transcription 3 (STAT3) phosphorylation, facilitating immune evasion (4, 13). Additionally, it promotes lactate-driven acidification of the tumor microenvironment and induces epigenetic modifications at the ATP-binding cassette sub-family B member 1 (ABCB1) promoter, maintaining cancer stemness and drug efflux capacity (4, 13). These metabolic functions, combined with regulatory roles in mitochondrial dynamics and redox homeostasis, establish PKM2 as a central metabolic hub in tumor adaptation.

### 2.2 Non-metabolic functions of PKM2 as a multifunctional hub

Beyond metabolism, PKM2 translocates to the nucleus under stress, where it phosphorylates histone H3 at threonine 11, activating c-Myc and Cyclin D1 expression and cooperating with HIF-1α to regulate the mTORC1 pathway, thus linking metabolic reprogramming to cell proliferation (4, 13, 14). In hepatocellular carcinoma, PKM2 is regulated via multi-level mechanisms including transcriptional activation by YAP through HIF-1α, post-translational modifications by HSP90 and GSK-3β that stabilize its dimeric conformation, and nuclear functions involving PRMT6 and STAT3 signaling to amplify aerobic glycolysis (15–18). PKM2’s SUMOylation promotes interaction with ARRDC1 and secretion via exosomes into the tumor microenvironment, activating STAT3 phosphorylation in monocytes and inducing their metabolic reprogramming and differentiation into macrophages (7). The exosomal circPETH-147aa further drives aerobic glycolysis via ALDOA-S36 phosphorylation, enhancing amino acid metabolic reprogramming and immune evasion (19). At the plasma membrane, TSP50 inhibits PKM2 activity via acetylation at K433, promoting HCC cell proliferation (20). PKM2 suppresses apoptosis by promoting Bim degradation, while its depletion stabilizes Bim and induces cell death (21). Moreover, PKM2 modulates immune escape by upregulating PD-L1 through STAT3 phosphorylation, recruiting HDAC3 to remodel chromatin accessibility, and sustaining oncogenic signaling through interactions with β-catenin and activation of CCND1 to accelerate the cell cycle (4, 14). These multifaceted non-metabolic functions position PKM2 as an integrative hub that connects metabolism, epigenetics, immune modulation, and cell cycle regulation in cancer progression.

## 3 Molecular mechanisms of PKM2-driven metabolic reprogramming

### 3.1 Glycolysis

PKM2 catalyzes the final, rate-limiting step of glycolysis by transferring a phosphate group from phosphoenolpyruvate (PEP) to adenosine diphosphate (ADP), generating pyruvate and adenosine triphosphate (ATP) (22). As a critical metabolic regulator, PKM2 undergoes dynamic structural transitions that enable dual functions. It facilitates the conversion of phosphoglycerate mutase (PGM)-derived intermediates into lactate and also acts as a protein kinase that participates in transcriptional regulation and metabolic reprogramming to sustain the Warburg effect (23). Post-translational modifications further modulate PKM2 activity. Phosphorylation at tyrosine 105 stabilizes the dimeric form and reduces pyruvate kinase activity, while hydroxylation at proline 403/408 enhances the expression of glucose transporter 1 (GLUT1) and lactate dehydrogenase A (LDHA) by activating hypoxia-inducible factor 1α (HIF-1α), reinforcing a positive feedback loop that maintains aerobic glycolysis (22, 24, 25). Nuclear translocation of PKM2, mediated by the extracellular signal-regulated kinase (ERK)/mitogen-activated protein kinase (MAPK) pathway, enables histone H3 phosphorylation and activation of c-Myc target genes. PKM2 also catalyzes phosphorylation of phosphoglycerate mutase 1 (PGAM1) at histidine 11, which enhances aerobic glycolysis and promotes tumor growth (6, 22, 26). The phosphoinositide 3-kinase (PI3K)/protein kinase B (Akt) pathway further supports this process by promoting LDHA-mediated conversion of pyruvate to lactate, contributing to tumor-specific accumulation of glycolytic end-products (27). In ovarian cancer, HIF-1α upregulates endothelial cell-specific molecule 1 (ESM1), which enhances PKM2 SUMOylation and stabilizes its dimeric form. This activates signal transducer and activator of transcription 5 (STAT5), forming a cycle that amplifies glucose uptake and lactate production (28). In lung cancer, PKM2 interacts with histone H2B and reduces its monoubiquitination (H2Bub1), thereby inhibiting the expression of mitochondrial respiration genes and promoting the Warburg effect (29). In triple-negative breast cancer, methyltransferase 14 (METTL14)-mediated N6-methyladenosine (m6A) modification facilitates PKM2 degradation through the miR-29c-3p/TRIM9 axis, shifting the balance toward the low-activity dimer (30). Meanwhile, crotonylation of polypyrimidine tract-binding protein 1 (PTBP1) at lysine 266 enhances heterogeneous nuclear ribonucleoproteins A1 and A2 (hnRNPA1/2) binding to PKM pre-mRNA, thereby promoting PKM2-specific splicing (31). In breast cancer, coactivator-associated arginine methyltransferase 1 (CARM1)-mediated methylation of PKM2 promotes its interaction with inositol 1,4,5-trisphosphate receptors (InsP3Rs), reducing endoplasmic reticulum to mitochondria Ca²^+^ flux and triggering aerobic glycolysis (32). In prostate cancer, long non-coding RNA (lncRNA) SNHG3 competitively binds to miR-139-5p and relieves its suppression of PKM2 mRNA, resulting in enhanced aerobic glycolysis (33). In non-small cell lung cancer (NSCLC), hypoxia-induced HIF-1α forms a complex with phosphorylated Smad3, which upregulates c-Myc and promotes PKM2 splicing, constructing a hypoxia-adaptive aerobic glycolysis network (34, 35). Notably, tyrosine phosphorylation at residues Y105 and Y148 exerts dual effects by maintaining PKM2 in its low-activity dimeric form while promoting Aldehyde Dehydrogenase-positive (ALDH^+^) cancer stem cell phenotypes (36). Despite its central role in glycolysis, the activity of PKM2 is modulated by cellular context through structural, epigenetic, and metabolic mechanisms. Structural remodeling, such as SUMOylation and O-linked β-N-acetylglucosamine (O-GlcNAcylation), stabilizes the dimeric conformation and adjusts enzymatic activity in response to environmental inputs (28, 37). Epigenetically, phosphorylation of hnRNPA1 at serine 6 enhances the recruitment of splicing factors, promoting the generation of the PKM2 isoform under specific regulatory cues (38–40). In parallel, PEP-dependent phosphorylation of PGAM1 facilitates the redirection of glycolytic intermediates into biosynthetic pathways, enabling cells to adjust to proliferative demands (26). In conclusion, these mechanisms allow PKM2 to serve as both a metabolic enzyme and a transcriptional modulator, with its function precisely tailored by tumor-specific microenvironmental signals and intracellular stress states.

**Figure 2:**
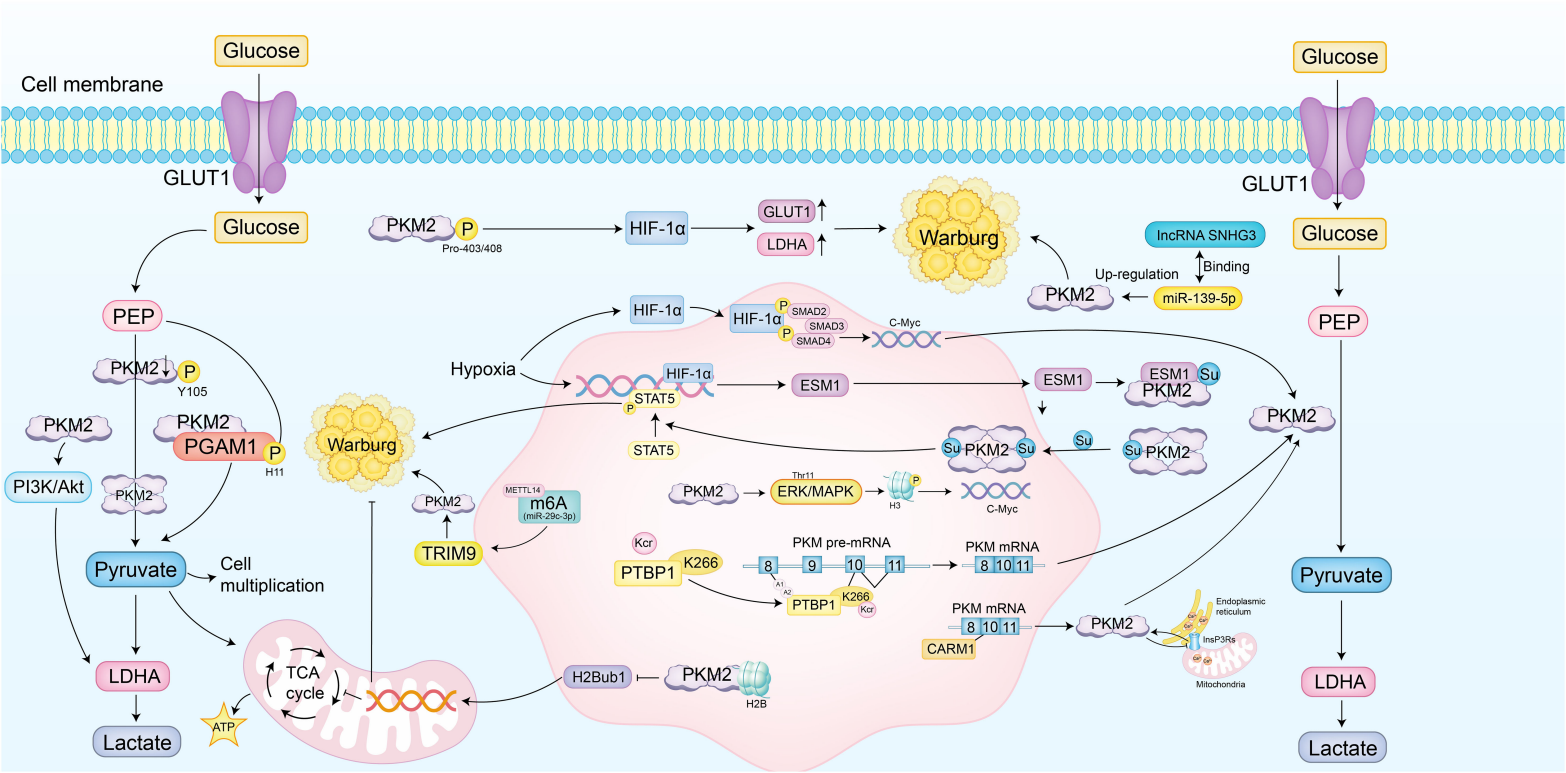
PKM2 drives metabolic reprogramming of glycolytic mechanisms. PKM2 catalyzes the final, rate-limiting step of glycolysis, converting phosphoenolpyruvate (PEP) to pyruvate and ATP. It also functions as a protein kinase involved in transcriptional regulation and metabolic reprogramming to sustain the Warburg effect. Post-translational modifications, such as phosphorylation at Tyr105, reduce its activity by stabilizing the dimeric form, while hydroxylation at Pro403/408 enhances the expression of glucose transporter 1 (GLUT1) and lactate dehydrogenase A (LDHA) via activation of hypoxia-inducible factor 1α (HIF-1α). Nuclear translocation of PKM2, mediated by the ERK/MAPK pathway, leads to histone H3 phosphorylation and activation of c-Myc target genes. PKM2 also phosphorylates phosphoglycerate mutase 1 (PGAM1) at His11, enhancing aerobic glycolysis. The PI3K/Akt pathway supports this process by promoting LDHA-mediated conversion of pyruvate to lactate. In ovarian cancer, HIF-1α upregulates endothelial cell-specific molecule 1 (ESM1), enhancing PKM2 SUMOylation and stabilizing its dimeric form, activating STAT5 and amplifying glucose uptake and lactate production. In lung cancer, PKM2 interacts with histone H2B, reducing its monoubiquitination (H2Bub1) and inhibiting mitochondrial respiration genes. In triple-negative breast cancer, N6-methyladenosine (m6A) modification by methyltransferase 14 (METTL14) facilitates PKM2 degradation, shifting towards the low-activity dimer. In breast cancer, methylation by coactivator-associated arginine methyltransferase 1 (CARM1) promotes PKM2 interaction with inositol 1,4,5-trisphosphate receptors (InsP3Rs), reducing endoplasmic reticulum to mitochondria Ca²^+^ flux. In prostate cancer, long non-coding RNA (lncRNA) SNHG3 relieves miR-139-5p suppression of PKM2 mRNA, enhancing aerobic glycolysis. In non-small cell lung cancer (NSCLC), hypoxia-induced HIF-1α forms a complex with phosphorylated Smad3, upregulating c-Myc and promoting PKM2 splicing. Structural remodeling, such as SUMOylation and O-GlcNAcylation, stabilizes the dimeric conformation and adjusts enzymatic activity. Epigenetic mechanisms, including phosphorylation of hnRNPA1, enhance the recruitment of splicing factors, promoting PKM2 isoform generation. PEP-dependent phosphorylation of PGAM1 redirects glycolytic intermediates into biosynthetic pathways, enabling cells to adjust to proliferative demands. These mechanisms allow PKM2 to serve as both a metabolic enzyme and a transcriptional modulator, with its function precisely tailored by tumor-specific microenvironmental signals and intracellular stress states.

These findings demonstrate that PKM2 serves as a central integrator of glycolytic regulation, shaped by structural remodeling, post-translational modifications, epigenetic control, and metabolic signaling. Multiple oncogenic pathways, including HIF-1α, MAPK, PI3K/Akt, and c-Myc, coordinately influence its oligomeric state, subcellular localization, and enzymatic activity. These regulatory inputs fine-tune PKM2 to sustain elevated aerobic glycolysis and enable cellular adaptation to hypoxia, nutrient fluctuations, and proliferative stress. By coupling metabolic output with transcriptional and post-transcriptional regulation, PKM2 functions not only as a metabolic enzyme but also as a signaling node that links energy metabolism to tumor progression (**Figure 2**).

### 3.2 Lipids

PKM2 orchestrates lipid metabolic reprogramming through structural transformation and signaling interactions that promote tumor progression. In the hypoxic microenvironment of ovarian cancer, HIF-1α upregulates endothelial cell-specific molecule 1 (ESM1), which induces PKM2 SUMOylation and stabilizes its dimeric conformation. This facilitates nuclear translocation of PKM2, enabling activation of fatty acid synthase (FASN) expression via STAT3 phosphorylation, thereby promoting *de novo* lipogenesis, tumor proliferation, and vasculogenic mimicry (28). This pro-oncogenic mechanism is amplified during peritoneal metastasis, where HIF-1α-induced PKM2 expression enhances both fatty acid uptake and lipid biosynthesis, establishing lipid metabolism as a core adaptive strategy in response to hypoxia and energy stress (41). In tumors associated with metabolic disorders, aerobic glycolysis-dominant PKM2 activity restricts pyruvate entry into mitochondria, suppressing oxidative phosphorylation (OXPHOS) and contributing to hepatic steatosis, which further reinforces a tumor-promoting metabolic niche (42). The lipid-regulatory role of PKM2 also exhibits context-dependent characteristics, particularly in response to therapeutic and metabolic stress. In cisplatin-resistant non-small cell lung cancer (NSCLC), PKM2 inhibition suppresses aerobic glycolysis and induces a compensatory increase in lipid metabolism through carnitine palmitoyltransferase 1A (CPT1A)-dependent fatty acid oxidation (FAO), sustaining cancer cell survival under chemotherapeutic pressure. This adaptive metabolic reprogramming can be reversed by Compound 3K, a PKM2 inhibitor that restores chemosensitivity (43, 44). In triple-negative breast cancer (TNBC), PKM2 upregulates acyl-CoA dehydrogenase very long chain (ACADVL) through the AMP-activated protein kinase–Krüppel-like factor 4 (AMPK–KLF4) axis, promoting lipid β-oxidation while depleting lipid droplet storage. This metabolic reprogramming concurrently suppresses aerobic glycolysis and enhances lipolysis, establishing a compensatory metabolic equilibrium that functions independently of BRCA mutation status (45). These findings highlight that PKM2 dynamically modulates lipid synthesis and degradation based on external stress conditions, underscoring its role in maintaining metabolic plasticity within diverse tumor microenvironments.

### 3.3 Amino acid

PKM2 reprograms amino acid metabolism by integrating enzymatic complex assembly with transcriptional control, thereby supporting tumor growth under metabolic stress. In triple-negative breast cancer (TNBC), the amino acid transporter SLC7A5 downregulates miR-152 and activates the E2F1/PTBP1 signaling axis, promoting alternative splicing of PKM pre-mRNA toward the PKM2 isoform. The increased expression of PKM2 enhances the uptake and utilization of essential amino acids, fueling biosynthetic demands and reinforcing tumor cell proliferation (46). This PKM2-driven shift also contributes to the emergence of drug-resistant metabolic phenotypes, highlighting its role in maintaining oncogenic adaptation through amino acid metabolic rewiring. Beyond its canonical glycolytic function, PKM2 exhibits a context-dependent regulatory role in response to amino acid deprivation. In the tumor microenvironment, where serine availability is limited, PKM2 coordinates with the c-Myc-responsive long non-coding RNA gLINC to assemble a metabolic enzyme complex comprising PGK1, PGAM1, ENO1, and LDHA. This complex significantly boosts aerobic glycolytic flux and enhances ATP production efficiency, enabling tumor cells to sustain energy output and survive serine-deficient stress (47, 48). These findings suggest that PKM2 facilitates metabolic flexibility not only through isoform control but also by structurally adapting to nutrient limitations, underscoring its dynamic role in amino acid-responsive metabolic reprogramming.

## 4 Specific regulatory network of PKM2 in digestive system tumors

While PKM2 is widely recognized for its role in glycolytic regulation, emerging evidence underscores a striking tissue-specific heterogeneity in its upstream modulation and functional outputs across digestive system tumors. In hepatocellular carcinoma, lncRNA DACT3-AS1 activates PKM2 via the HDAC2/FOXA3 axis, thereby promoting immune evasion and metastasis (49). In gastric cancer, the CCAT1–PTBP1 axis facilitates alternative splicing to favor PKM2 isoform dominance, promoting metabolic reprogramming and cancer stemness (50). In colorectal cancer, OTUB2-mediated deubiquitination prevents PKM2 degradation by interfering with Parkin, sustaining aerobic glycolysis under metabolic stress (51). In pancreatic ductal adenocarcinoma, TGF-β1 derived from tumor-associated macrophages induces PKM2 nuclear translocation and enhances STAT1-mediated PD-L1 transcription, linking glucose metabolism to immune escape (8). These findings reveal PKM2 as a tumor-context–sensitive integrator of metabolic, immunological, and epigenetic signals. Understanding these distinct regulatory circuits is critical for advancing tumor-specific metabolic therapies. The following sections will detail the regulatory networks of PKM2 in individual digestive cancers, highlighting both shared principles and unique adaptations.

### 4.1 Hepatocellular carcinoma

PKM2 exerts cancer-promoting effects in HCC through multi-level and multi-mechanistic regulation. In the hypoxic microenvironment, Yes-associated protein (YAP) maintains the stability of the interaction between HIF-1α and the PKM2 gene, directly activating PKM2 transcription and accelerating aerobic glycolysis (15). Glypican-3 promotes the metabolic reprogramming shift to aerobic glycolysis by upregulating PKM2 via HIF-1α (16). Nuclear-translocated PKM2 enhances aerobic glycolysis through Protein Arginine Methyltransferase 6 (PRMT6) and activates the STAT3 signaling pathway, persistently amplifying glycolytic flux (17). Post-translational modifications also strengthen the Warburg effect; specifically, Heat Shock Protein 90 (HSP90) and Glycogen Synthase Kinase 3 Beta (GSK-3β) cooperatively phosphorylate PKM2 at Thr-328, stabilizing its dimeric conformation (18). Under hypoxia, the RNA-binding protein HuR suppresses miR-199a, leading to increased PKM2 expression, which acts as a crucial switch for the Warburg effect (52). Likewise, the circMAT2B/miR-338-3p axis enhances PKM2 stability and expression under hypoxic conditions (53). Rhubarb extract and its active compound Rhein upregulate PKM2 expression, promoting aerobic glycolysis, though Rhein may exacerbate liver injury (54). A Methyltransferase-like 5 (METTL5) activates PKM2 transcription by upregulating Ubiquitin Specific Protease 5 (USP5) to inhibit c-Myc ubiquitin-mediated degradation (55), while downregulation of GATA6 drives metabolic reprogramming in HCC cells (56). Non-canonical functions of PKM2 include its SUMOylation, which promotes interaction with ARRDC1 and secretion via exosomes into the tumor microenvironment, activating STAT3 phosphorylation in monocytes and inducing their metabolic reprogramming and differentiation into macrophages (7). The exosomal circular RNA circPETH-147aa promotes aerobic glycolysis via ALDOA-S36 phosphorylation, driving amino acid metabolic reprogramming and immune evasion (19). At the plasma membrane, testis-specific protease 50 (TSP50) inhibits PKM2 activity through acetylation at K433, promoting HCC cell proliferation (20). Within metabolic interaction networks, Gankyrin activates the β-catenin/c-Myc axis to upregulate PKM2 expression, strengthening the connection between glucose and glutamine metabolism and accelerating tumor progression (57). In HCC associated with type Ia glycogen storage disease, G6Pase-α deficiency leads to PKM2 upregulation, promoting aerobic glycolysis and the hexose monophosphate shunt, thereby accelerating tumor development (58). HDAC8-mediated deacetylation of PKM2 at K62 facilitates its nuclear translocation, where it binds β-catenin and activates CCND1 to accelerate the cell cycle (59). Furthermore, PKM2 suppresses apoptosis by promoting Bim degradation; PKM2 depletion stabilizes Bim and induces cell death (21). Clinical studies confirm that PKM2 exerts its oncogenic role in HCC by downregulating MicroRNA-122 (miR-122); miR-122 directly targets the 3’UTR of PKM2, and restoration of miR-122 expression suppresses glucose uptake and tumor growth (60).

**Figure 3:**
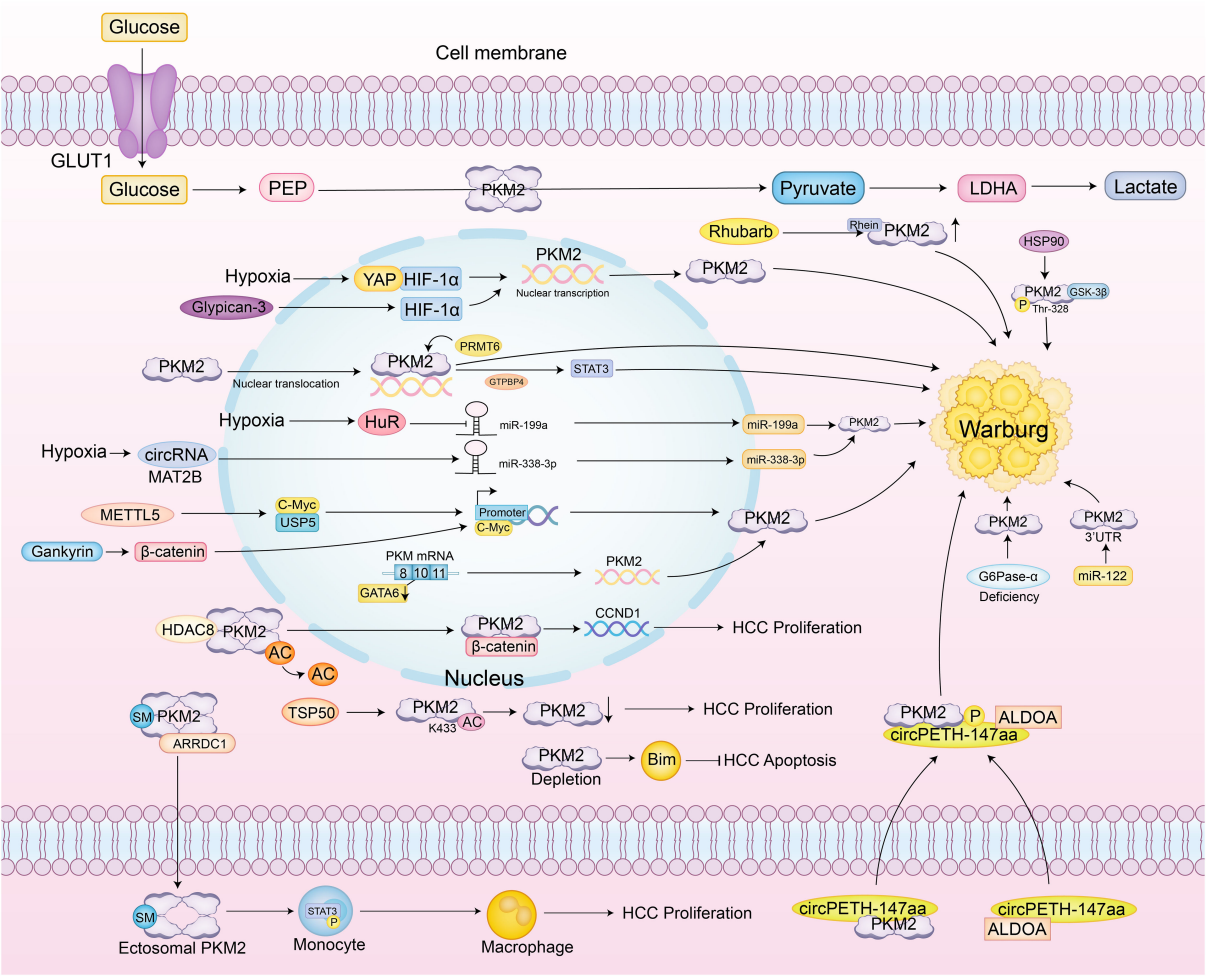
Specific regulatory network of PKM2 in hepatocellular carcinoma. PKM2 catalyzes the conversion of phosphoenolpyruvate (PEP) to pyruvate, which is further converted to lactate, a key aspect of the Warburg effect observed in cancer cells. Under hypoxic conditions, glycogen synthase kinase 3 beta (GSK-3β) and heat shock protein 90 (HSP90) phosphorylate PKM2 at Thr-328, stabilizing its dimeric form and enhancing aerobic glycolysis. Yes-associated protein (YAP) and hypoxia-inducible factor 1-alpha (HIF-1α) interact to directly activate PKM2 transcription, further promoting glycolysis. The RNA-binding protein HuR suppresses miR-199a under hypoxia, leading to increased PKM2 expression. Additionally, the circMAT2B/miR-338-3p axis enhances PKM2 stability and expression under hypoxic conditions. Methyltransferase-like 5 (METTL5) activates PKM2 transcription by upregulating Ubiquitin Specific Protease 5 (USP5), which inhibits c-Myc ubiquitin-mediated degradation. PKM2 also undergoes SUMOylation, promoting its interaction with ARRDC1 and secretion via exosomes into the tumor microenvironment, where it activates STAT3 phosphorylation in monocytes, inducing their metabolic reprogramming and differentiation into macrophages. The exosomal circular RNA circPETH-147aa promotes glycolysis via ALDOA-S36 phosphorylation, driving amino acid metabolic reprogramming and immune evasion. At the plasma membrane, testis-specific protease 50 (TSP50) inhibits PKM2 activity through acetylation at K433, promoting HCC cell proliferation. Gankyrin activates the β-catenin/c-Myc axis to upregulate PKM2 expression, strengthening the connection between glucose and glutamine metabolism and accelerating tumor progression. In HCC associated with type Ia glycogen storage disease, G6Pase-α deficiency leads to PKM2 upregulation, promoting aerobic glycolysis and the hexose monophosphate shunt, thereby accelerating tumor development. HDAC8-mediated deacetylation of PKM2 at K62 facilitates its nuclear translocation, where it binds β-catenin and activates CCND1 to accelerate the cell cycle. PKM2 also suppresses apoptosis by promoting Bim degradation; PKM2 depletion stabilizes Bim and induces cell death. Clinical studies confirm that PKM2 exerts its oncogenic role in HCC by downregulating MicroRNA-122 (miR-122); miR-122 directly targets the 3’UTR of PKM2, and restoration of miR-122 expression suppresses glucose uptake and tumor growth.

PKM2 also exhibits clear context-dependent effects in HCC. When Ser333 is unphosphorylated, PKM2 promotes tumor growth, whereas ULK1-mediated phosphorylation at Ser333 enhances PKM2 enzymatic activity, reduces nuclear localization, suppresses c-Myc expression, and attenuates the Warburg effect, demonstrating an inhibitory role dependent on context (61). High PKM2 expression predicts poor prognosis and inhibits apoptosis by promoting Bim degradation, while PKM2 knockdown stabilizes Bim and induces apoptosis, indicating that its cancer-promoting effect depends on its interaction with apoptotic regulators (21). Additionally, COX-2 and PKM2 are both elevated in HCC and correlate with poor prognosis. Knockdown of COX-2 reduces PKM2 and HIF-1α expression, inhibiting proliferation and increasing apoptosis. However, PKM2 inhibition increases apoptosis without altering COX-2 or HIF-1α levels, suggesting that PKM2’s effects rely on upstream COX-2/HIF-1α signaling (62) (**Figure 3**).

### 4.2 Gastric cancer

**Figure 4:**
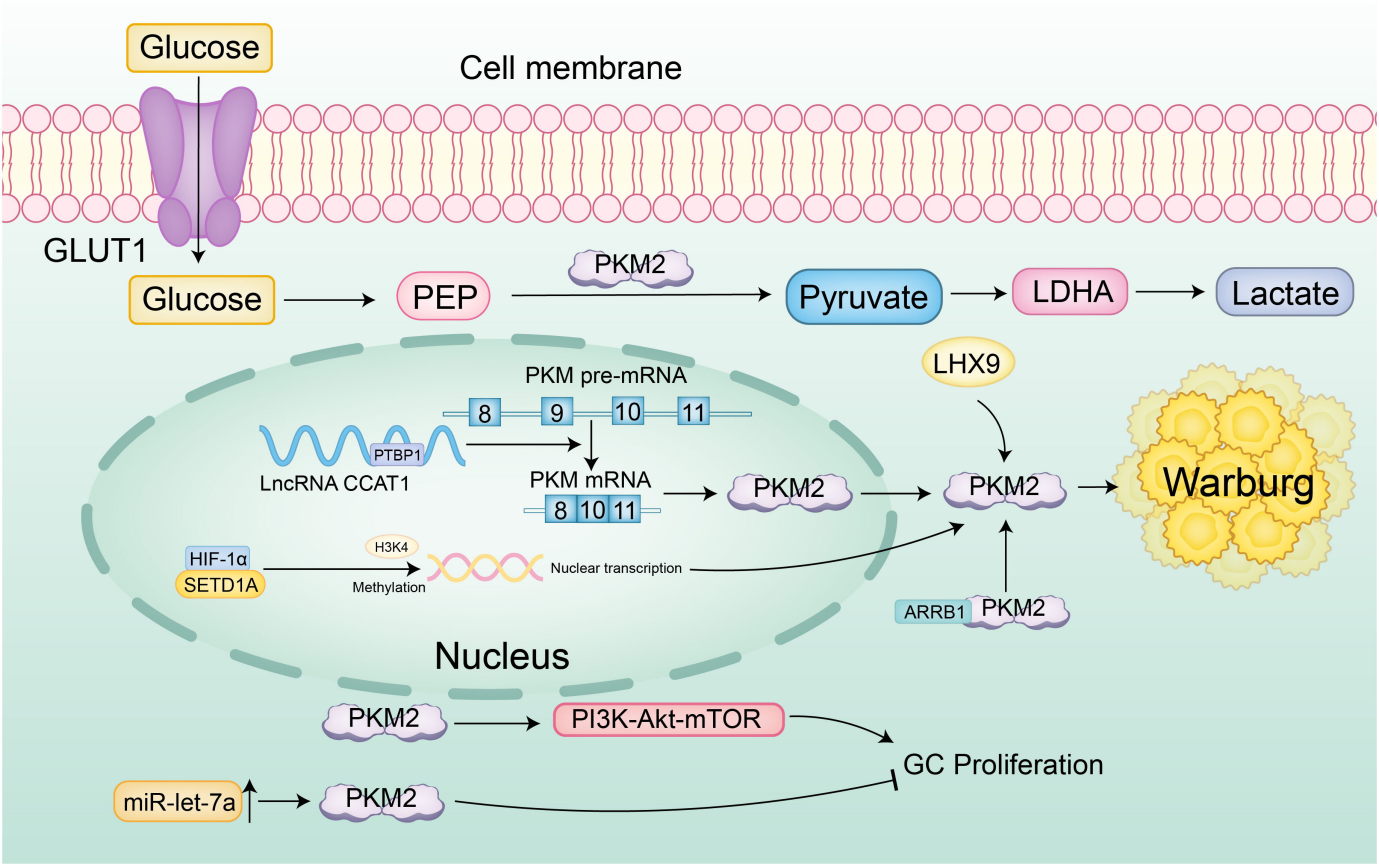
Specific regulatory network of PKM2 in gastric cancer. Glucose is transported into cells via GLUT1 and converted to pyruvate by PKM2, which is then metabolized to lactate, a hallmark of the Warburg effect. The long non-coding RNA (lncRNA) CCAT1 promotes the alternative splicing of PKM pre-mRNA towards the low-activity PKM2 isoform by stabilizing PTBP1, resulting in the accumulation of dimeric PKM2 and reduced enzymatic activity. Additionally, SETD1A enhances HIF-1α recruitment to the PKM2 promoter through H3K4 methylation, forming a positive feedback loop that persistently amplifies aerobic glycolysis. β-Arrestin 1 (ARRB1) binds PKM2, inhibiting its tetramer assembly and maintaining a low-activity dimeric state that promotes the Warburg effect. LIM Homeobox 9 (LHX9) activates PKM2’s catalytic function, driving metabolic reprogramming and malignant phenotypes in gastric cancer stem cells. Reduced PKM2 expression may weaken PI3K-Akt-mTOR signaling, activating autophagy and reducing the migratory capacity of gastric cancer cells. Furthermore, overexpression of miR-let-7a suppresses proliferation, migration, and invasion of gastric cancer cells by downregulating PKM2, highlighting the context-dependent regulation of PKM2’s oncogenic potential.

At the level of transcriptional splicing, the long non-coding RNA (lncRNA) CCAT1 facilitates alternative splicing of PKM pre-mRNA towards the low-activity PKM2 isoform by binding and stabilizing PTBP1 protein, resulting in dimeric PKM2 accumulation that lowers enzymatic activity. This causes glycolytic intermediates to accumulate, diverting metabolic flux toward aerobic glycolysis, thereby markedly increasing lactate production and glucose flux and establishing a pro-oncogenic metabolic reprogramming phenotype (50). Aerobic glycolysis is also precisely regulated at the epigenetic level-histone lysine methyltransferase SETD1A enhances HIF-1α recruitment to the PKM2 promoter through Histone H3 Lysine 4 (H3K4) methylation, forming a HIF-1α/SETD1A positive feedback loop that persistently amplifies glycolytic flux and sustains the continuous proliferation of gastric cancer cells (63). At the enzymatic regulation level, PKM2’s role is more nuanced and context-dependent. β-Arrestin 1 (ARRB1) directly binds PKM2 and inhibits its tetramer assembly, maintaining a low-activity dimeric state that promotes the Warburg effect. In contrast, LIM Homeobox 9 (LHX9) activates PKM2’s catalytic function, driving metabolic reprogramming and malignant phenotypes in gastric cancer stem cells, effects that can be reversed by LHX9 knockdown (64, 65). Additionally, cytoplasmic PKM2 exhibits non-canonical functions; reduced PKM2 expression may weaken PI3K-Akt-mTOR signaling, activating autophagy and reducing the migratory capacity of gastric cancer cells (66, 67). Overexpression of miR-let-7a suppresses proliferation, migration, and invasion of gastric cancer cells by downregulating PKM2, further illustrating the context-dependent regulation of PKM2’s oncogenic potential (68) (**Figure 4**).

### 4.3 Colorectal cancer

In colorectal cancer, PKM2 plays a prominent cancer-promoting role by enhancing glycolytic flux and supporting tumor progression. The deubiquitinase OTUB2 directly binds to PKM2, preventing its interaction with the E3 ubiquitin ligase Parkin, thereby stabilizing PKM2 and enhancing its enzymatic activity by blocking ubiquitination and degradation. Activated PKM2 promotes aerobic glycolysis, markedly increasing glucose consumption and lactate production, particularly under glucose-starved conditions, where this pathway reinforces tumor cell dependence on aerobic glycolysis (51). At the transcriptional level, the 53-amino-acid peptide encoded by HOXB-AS3 binds to the RGG motif of hnRNP A1, preventing its interaction with PKM exon 9 and thereby inhibiting the splicing of PKM into the PKM2 isoform, reducing PKM2 levels and suppressing glucose metabolic reprogramming (69). In early tumorigenesis, PKM2 is already overexpressed and cooperates with HIF-1α and GLUT1 to activate the glycolytic program, establishing an early Warburg effect axis that provides metabolic advantages to emerging tumor cells (70). Epigenetically, PRL-3 promotes primary tumor proliferation and metastatic capacity by upregulating PKM2 and glycolytic enzymes such as Glut1, HK2, and LDHA, collectively enhancing glucose uptake and lactate production (71).

The functional output of PKM2 in colorectal cancer also exhibits notable context-dependent effects. The tumor suppressor NDRG2 inhibits metabolic reprogramming through a dual mechanism: it directly reduces PKM2 expression, limiting pyruvate production, while concurrently inhibiting c-Myc transcriptional activity, which in turn suppresses GLUT1- and HK2-mediated glucose uptake and phosphorylation (72). In metastasis regulation, activated YAP drives Glut3 expression and recruits PKM2 to synergistically enhance the transcriptional activation of glycolytic genes, promoting tumor invasiveness and stem-like properties in a feed-forward loop (73). However, during liver metastasis, this aerobic glycolysis-driven phenotype is modulated by PKLR, which reprograms glutathione metabolism to maintain redox homeostasis by negatively regulating PKM2 activity. Inhibition of PKLR disrupts this adaptive metabolic balance and significantly impairs the liver colonization ability of colorectal cancer cells (74).

### 4.4 Pancreatic cancer

At the transcriptional level, the lncRNA MIR210HG enhances glycolytic flux and promotes cancer cell proliferation and invasion by sponging miR-125b-5p to relieve its suppression on PKM2 and HK2. Knockout of MIR210HG reverses this phenotype, confirming the regulatory significance of the MIR210HG/miR-125b-5p/PKM2 axis (75). PKM2 also participates in energy production through atypical lactate metabolic pathways, as indicated by the abnormal upregulation of lactate dehydrogenase-B, reflecting broader metabolic reprogramming (76). In signaling pathway interactions, PKM2 activates the NF-κB/p65 pathway to upregulate HIF-1α expression and transcriptional activity and induces VEGF-A secretion to promote tumor angiogenesis. PKM2 deficiency impairs NF-κB signaling, reduces angiogenesis, and increases apoptosis (77). Functionally, PKM2 contributes to chemoresistance by suppressing p38-mediated phosphorylation of p53 at serine 46 and by inhibiting caspase 3/7 and PARP cleavage in response to gemcitabine treatment (78). In pancreatic ductal adenocarcinoma, PKM2 knockdown markedly reduces proliferation, migration, and tumorigenic potential, supporting its role as a core oncogenic driver (79). In metastasis regulation, PKM2 enhances cancer cell migration by stabilizing PAK2 protein through phosphorylation. Silencing PKM2 accelerates PAK2 degradation, disrupts tumor–stellate cell interactions, and inhibits the epithelial–mesenchymal transition process (80, 81).

The oncogenic functions of PKM2 in pancreatic cancer also demonstrate context-dependent characteristics, particularly under microenvironmental and metabolic constraints. Tumor-associated macrophage-derived TGF-β1 induces nuclear translocation of PKM2 and promotes its interaction with STAT1, which activates the PD-L1 promoter and drives immune checkpoint expression. PKM2 knockdown restores natural killer cell cytotoxicity and reverses immune evasion, indicating that its immunomodulatory effects depend on inflammatory signals within the tumor microenvironment (8). Under metabolic stress conditions, PKM2 sustains the Warburg effect by maintaining glucose uptake and lactate production. Its silencing suppresses aerobic glycolysis and activates caspase-3/7, thereby impairing cell survival and invasive capacity. These findings highlight that PKM2-mediated metabolic advantages are tightly linked to environmental nutrient availability (82).

### 4.5 Esophageal cancer

At the fundamental metabolic level of esophageal squamous cell carcinoma, ESRRG inhibits the transcriptional expression of PKM2 by directly binding to its promoter, thereby reducing glycolytic activity and blocking cell proliferation. Downregulation of ESRRG expression releases this inhibitory effect, resulting in abnormal PKM2 upregulation and enhanced lactate metabolism, which collectively establish a pro-cancer metabolic phenotype (83). In the context of treatment resistance, high PKM2 expression reduces cisplatin sensitivity by sustaining the activity of the pentose phosphate pathway, and its inhibition disrupts pyruvate kinase function, leading to a surge in ROS levels and imbalance of the NADPH/NADP ratio, thereby reversing chemotherapy resistance (84). In the oxidative stress response, the oncogenic effect of PKM2 is closely shaped by the surrounding regulatory state. Activation of Nrf2 promotes PKM2 oligomerization by inducing its glycosylation modification, driving metabolic reprogramming that supports tumor progression. However, this effect remains dependent on a functional glycolytic program, as specific glycolysis inhibition can effectively block the proliferation of esophageal squamous cell carcinoma cells exhibiting high Nrf2 activity, suggesting that PKM2 functions in a context-dependent manner under redox-sensitive conditions (85).

### 4.6 Oral squamous cell carcinoma

In oral squamous cell carcinoma, the circadian rhythm gene TIMELESS promotes tumor progression through a SIRT1-mediated metabolic axis. TIMELESS upregulates SIRT1, which activates glycolytic enzymes including PKM2, HK2, LDHA, and GLUT1. This pathway enhances glycolytic activity by increasing glucose uptake and lactate production, ensuring a continuous energy supply under hypoxic conditions and supporting tumor cell proliferation and survival (86) (**Table 1**).

**Table 1:** Specific regulatory network of PKM2 in digestive system tumors.

Tumor Type	Upstream Regulators	Downstream Pathways	Non-Canonical Functions	Unique Regulatory Patterns	References
Hepatocellular carcinoma	YAP, HIF-1α, circMAT2B, METTL5, miR-122	STAT3, β-catenin/CCND1, Warburg effect	Exosomal PKM2-STAT3 signaling in monocytes; Bim degradation	G6Pase-α deficiency; TSP50-K433 acetylation	(20, 58)
Gastric Cancer	CCAT1, SETD1A, LHX9, miR-let-7a	PI3K-AKT-mTOR, HIF-1α amplification	Regulation of autophagy via PKM2	LHX9-mediated PKM2 activation; H3K4 methylation-driven feedback	(63, 65)
Colorectal Cancer	OTUB2, HOXB-AS3, PRL-3, YAP	GLUT1, HK2, LDHA, c-Myc	Redox reprogramming via PKLR; metastasis regulation	HOXB-AS3 blocks PKM2 splicing; YAP recruits PKM2 for feedforward glycolysis	(69, 73)
Pancreatic Cancer	MIR210HG, miR-125b-5p, PAK2	NF-κB/p65 → HIF-1α → VEGF-A, p53-S46	Immune evasion via PD-L1 (STAT1) induction	TGF-β1-driven PKM2 nuclear translocation; PKM2-PAK2 stabilizing axis	(8, 81)
Esophageal Cancer	ESRRG, Nrf2, cisplatin	NADPH/NADP balance, pentose phosphate pathway	Glycosylation-mediated PKM2 oligomerization	Nrf2-induced glycosylation of PKM2; ESRRG inhibition → PKM2	(83, 85)
oral squamous cell carcinoma	TIMELESS → SIRT1	PKM2/HK2/LDHA/GLUT1	Circadian rhythm–linked metabolic reprogramming	TIMELESS–SIRT1–PKM2 axis under hypoxia	(86)

## 5 Innovative therapeutic strategies targeting PKM2

### 5.1 Small molecule inhibitors and other metabolic interventions

PKM2-specific small molecule inhibitors exert antitumor effects by directly targeting its enzymatic activity or regulating its structural conformation. Isoacteoside binds to the PKM2 active site, inhibits its catalytic function, and synergistically enhances the antitumor efficacy of sorafenib in hepatocellular carcinoma (87). Among natural compounds, euphorbia factor L3 and ellagic acid act as competitive inhibitors, while curcumin and resveratrol function as non-competitive inhibitors by disrupting metabolic complex formation. Ellagic acid demonstrates the strongest anticancer activity among these (88). An irreversible inhibitor, N-(4-(3-(3-(methylamino)-3-oxo-propyl)-5-(4′-(trifluoromethyl)-[1,1’-biphenyl]-4-yl)-1H-pyrazol-1-yl)phenyl)propionamide, covalently binds to PKM2 at Cys326/317, selectively inhibiting its kinase activity and destabilizing the protein, thus suppressing glycolysis without affecting PKM1 (89). Shikonin reverses chemotherapy resistance caused by SIRT1 deficiency and restores oxaliplatin sensitivity in colorectal cancer by targeting PKM2 (90, 91). Tanshinone II.A upregulates miR-122, downregulates PKM2, blocks glycolysis, and induces cell cycle arrest in esophageal cancer (92). In non-small cell lung cancer, casticin and Coenzyme Q0 (CoQ0) suppress HIF-1α signaling, leading to reduced PKM2 expression, inhibition of glucose metabolism, and reversal of the Warburg effect (93, 94). These two mechanisms share similarities with the HIF-1α/PKM2 positive feedback loop observed in liver cancer.

Therapeutic modulation of PKM2 function can also be achieved through structural reprogramming. In gastric cancer, the PKM2 activator DASA-58 overcomes ARRB1-mediated tetramerization inhibition, restoring pyruvate kinase activity and suppressing tumor growth (64). Butyrate promotes PKM2 dephosphorylation and tetramer formation, suppresses the Warburg effect, and alters nucleotide metabolism to restore homeostasis in colorectal cancer (95). Metformin inhibits PKM2 via both AMP-activated protein kinase (AMPK)-dependent and -independent mechanisms, reducing FASN/HK2 expression through c-Myc suppression and directly impairing ATP production (96). Targeting splicing regulators provides an alternative strategy. The HOXB-AS3 peptide blocks hnRNPA1 from binding PKM pre-mRNA, suppressing PKM2 isoform generation (69). Similarly, miRNAs modulate splicing factor activity to promote PKM1-dominant expression, reversing glycolytic phenotypes (97). In hepatocellular carcinoma, ZFP91 promotes hnRNPA1 ubiquitination and inhibits PKM2 splicing (98), while SIRT1/6 inhibitors regulate hnRNPA1 acetylation to control PKM2 expression (99). Several compounds target PKM2-interacting proteins to modulate its activity. TRIM35 inhibits Y105 phosphorylation, thereby suppressing the Warburg effect (100); SULT2B1 inhibitors block the AKT/PKM2 axis to reduce glycolysis (101); and PRDX2 inhibitors prevent PKM2 nuclear translocation, attenuating STAT3 signaling activation (102). PKM2 agonists that bind allosteric pockets distal from the FBP site divert metabolic intermediates away from serine biosynthesis, indirectly promoting serine generation to support tumor proliferation (103) (**Tables 2**, **3**).

**Table 2:** Small molecule inhibitors.

Therapeutic Strategy	Mechanism of Action	Stage	References
Isoacteoside	Inhibits PKM2 activity	Preclinical stage	(87)
Euphorbia factor L1, Ellagic acid	Competitively inhibits PKM2 catalytic function	Preclinical stage	(88)
Curcumin, Resveratrol	Non-competitively inhibits metabolic complex formation	Preclinical stage	(88)
Novel irreversible inhibitors	Covalently binds to PKM2 at Cys326/317, inhibiting PKM2 activity without affecting PKM1	Preclinical stage	(89)
Shikonin	Inhibits PKM2 to reverse chemotherapy resistance caused by SIRT1 deficiency	Preclinical stage	(90, 91)
Tanshinone II.A	Upregulates miR-122 to suppress PKM2 expression	Preclinical stage	(92)
Casticin	Targets HIF-1α to downregulate PKM2 expression	Preclinical stage	(94)
CoQ0	Inhibits HIF-1α expression, downregulating PKM2	Preclinical stage	(93)

**Table 3:** Other metabolic interventions.

Therapeutic Strategy	Mechanism of Action	Stage	References
DASA-58	Reverses ARRB1-mediated suppression of PKM2 tetramerization, restoring pyruvate kinase activity	Preclinical stage	(64)
Butyrate	Promotes PKM2 dephosphorylation and tetramerization, inhibits the Warburg effect, and reduces nucleotide levels	Preclinical stage	(95)
Metformin	Inhibits PKM2 activity through both AMPK-dependent and -independent pathways	Clinical stage/Approved drug	(96)
HOXB-AS3 peptide	Blocks binding of hnRNPA1 to PKM pre-mRNA, inhibiting PKM2 production	Preclinical stage	(69)
ZFP91	Regulates hnRNPA1 ubiquitination to inhibit PKM2 splicing	Preclinical stage	(98)
SIRT1/6 inhibitors	Modulates hnRNPA1 acetylation levels to regulate PKM2 expression	Preclinical stage	(99)
TRIM35	Inhibits Y105 phosphorylation of PKM2 to block the Warburg effect	Preclinical stage	(100)
SULT2B1 inhibitors	Disrupts the AKT/PKM2 signaling axis to inhibit glycolysis	Preclinical stage	(101)
PRDX2 inhibitors	Blocks PKM2 nuclear translocation and STAT3 signaling activation	Preclinical stage	(102)
PKM2 agonists	Binds to a pocket distant from the FBP site, redirecting glycolytic intermediates toward serine synthesis pathway	Preclinical to early clinical stage	(103)

### 5.2 Combination therapeutic strategies

Combination therapies targeting PKM2 alongside other metabolic or signaling pathways have shown enhanced efficacy. In pancreatic cancer, inhibition of mitochondrial uncoupling protein 2 (UCP2) by genipin enhances the efficacy of 2-deoxyglucose (2-DG), suggesting that co-targeting the UCP2–PKM2 metabolic axis disrupts mitochondrial bioenergetics and glycolysis simultaneously (104). In 5-fluorouracil (5-FU)-resistant colorectal cancer, suppression of PKM2 leads to upregulation of pyruvate kinase M1 (PKM1) and impairment of the pentose phosphate pathway (PPP), resulting in decreased nicotinamide adenine dinucleotide phosphate (NADPH) production and weakened antioxidant defenses. Combined inhibition of OXPHOS further blocks energy compensation, suppresses cancer stemness, and restores drug sensitivity (105). Targeting redox balance through dual metabolic inhibition is also effective. The glyoxalase I (GLO I) inhibitor TLSC702 increases cellular respiratory dependence, while shikonin inhibits PKM2 activity. This combination induces methylglyoxal accumulation, ATP depletion, and apoptosis, effectively blocking the glycolysis–OXPHOS metabolic switch (106). In colorectal cancer, combined inhibition of estrogen signaling and PKM2 reduces glucose uptake, increases reactive oxygen species (ROS) levels, and triggers apoptotic cell death (107). Other combinations interfere with PKM2-dependent transcriptional signaling. DASA-58 promotes the tetrameric conformation of PKM2, thereby preventing its nuclear translocation. When used with metformin, an OXPHOS inhibitor, this approach disrupts metastasis driven by cancer-associated fibroblasts (CAFs) and targets both glycolytic flux and mitochondrial respiration (108). In tumors retaining wild-type tumor protein p53 (TP53), activation of circular RNA FRMD4A (circFRMD4A) suppresses PKM2 expression. Co-treatment with the copper ionophore elesclomol induces copper-dependent cell death (cuproptosis), offering a mechanism-based strategy for overcoming chemotherapy resistance in digestive system tumors (109).

Although no clinical trials directly targeting PKM2 have yet advanced to the registration stage in digestive system tumors, multiple preliminary mechanistic studies and animal model validations have provided a solid foundation for subsequent human trial design. This warrants further exploration and advancement across various digestive system malignancies in future research (**Table 4**).

**Table 4:** Combination therapy strategies.

Therapeutic Combination	Mechanism of Action	Stage	References
Genipin + 2-DG	Targets the UCP2-PKM2 axis to enhance metabolic intervention efficacy in pancreatic cancer	Preclinical stage	(104)
E2 signaling inhibition + PKM2 blockade	Reduces glucose uptake, inhibits ROS generation, and increases apoptosis rate	Preclinical stage	(107)
TLSC702 + Shikonin	Blocks the glycolysis-mitochondrial respiration compensatory switch	Preclinical stage	(106)
PKM2 inhibition + OXPHOS inhibition	Upregulates PKM1 to suppress the pentose phosphate pathway, synergistically blocking energy compensation in 5-FU-resistant cells	Preclinical stage	(105)
DASA-58 + Metformin	Forces PKM2 tetramerization to inhibit nuclear translocation, combined with OXPHOS inhibition to block CAF-induced metastasis	Preclinical stage	(108)
p53/circFRMD4A + Elesclomol	Activates circFRMD4A to inhibit PKM2, inducing cuproptosis to suppress digestive system tumor growth	Preclinical stage	(109)

### 5.3 Clinical translation challenges

The clinical translation of PKM2-targeted therapies faces several multilayered challenges spanning biological, pharmacological, and regulatory domains. At the mechanistic level, the dual roles of PKM2 in cancer and immune regulation complicate therapeutic development. Beyond its function in tumor metabolism, PKM2 modulates TCF1^+^ CD8^+^ T cell activity via the PKM2–pentose phosphate pathway (PPP) axis. This immunometabolic crosstalk necessitates careful therapeutic design to balance tumor suppression with the preservation of immune effector function (110). The tumor microenvironment further complicates therapeutic outcomes. In pancreatic cancer, PKM2 inhibition under glucose-limited conditions paradoxically enhances cell survival, suggesting that nutrient availability can reprogram cellular responses to PKM2-targeted therapies (111). The tumor microenvironment further complicates therapeutic outcomes. In pancreatic cancer, PKM2 inhibition under glucose-limited conditions paradoxically enhances cell survival, suggesting that nutrient availability can reprogram cellular responses to PKM2-targeted therapies. Therefore, metabolic context must be considered to prevent adaptive resistance (112). Diagnostic-therapeutic integration also remains limited. The PKM2-targeted PET tracer Fluorine F 18 DASA-23 shows potential as an activator in glioblastoma imaging; however, its restricted blood-brain barrier permeability limits both diagnostic sensitivity and potential therapeutic extension (113).In conclusion, these challenges emphasize the need for context-dependent intervention strategies, rigorous preclinical validation, and early-phase clinical trial designs that integrate tumor metabolism, immune modulation, and pharmacodynamics into comprehensive evaluation frameworks.

Tumor cells often exploit metabolic plasticity to evade therapeutic interventions targeting PKM2. In glucose-limited microenvironments, digestive system tumors may switch from glycolysis to mitochondrial OXPHOS or FAO, thereby diminishing the efficacy of PKM2 inhibitors and paradoxically enhancing cell survival (43). For instance, in 5-FU-resistant colorectal cancer, PKM2 suppression leads to upregulation of PKM1 and impairment of the pentose phosphate pathway, which reduces NADPH and weakens redox defenses. However, cells compensate via increased OXPHOS dependency, a vulnerability that can be exploited through combined PKM2 and mitochondrial inhibition (64). Similarly, shikonin-mediated PKM2 inhibition induces glycolytic collapse, but cancer cells activate mitochondrial respiration unless this pathway is simultaneously blocked by agents such as TLSC702 (64). In cisplatin-resistant tumors, PKM2 inhibition activates CPT1A-dependent FAO, maintaining ATP production and conferring chemoresistance, which is reversible by dual-targeting metabolic regulators (44). These insights underscore the importance of developing therapeutic strategies that account for metabolic compensation by concurrently targeting glycolytic and compensatory energy pathways to circumvent resistance.

PKM2 plays a paradoxical role in immune regulation by both promoting tumor immune evasion and influencing T cell fate. On the one hand, nuclear PKM2 upregulates PD-L1 expression through STAT3 phosphorylation and remodels chromatin accessibility via HDAC3 recruitment, contributing to immunosuppressive tumor microenvironments (4). Lactate accumulation further reinforces this state by impairing T cell function and sustaining regulatory macrophage phenotypes (4). On the other hand, PKM2 also modulates CD8^+^ T cell differentiation. Its deficiency activates the pentose phosphate pathway and promotes the expansion of TCF1^+^ progenitor CD8^+^ T cells, which are essential for durable responses to immune checkpoint blockade (110). This duality presents a therapeutic dilemma: while PKM2 inhibition may benefit anti-tumor immunity via T cell reprogramming, it might concurrently impair metabolic homeostasis or drive adaptive resistance in the tumor. Additionally, agents such as PRDX2 inhibitors selectively block PKM2 nuclear translocation without abolishing its cytosolic functions, while preserving glycolytic support for T cells (102).

## 6 Conclusion

PKM2 functions as a central regulator of tumor metabolic reprogramming, orchestrating glycolysis, lipid synthesis, and amino acid metabolism to promote cancer cell proliferation, metastasis, and therapeutic resistance. Its regulatory influence spans the full spectrum of tumor initiation and progression, particularly in digestive system malignancies.

In carbohydrate metabolism, PKM2 inhibits mitochondrial oxidative phosphorylation via dynamic structural transitions and reinforces aerobic glycolysis by interacting with HIF-1α and c-Myc, thereby amplifying the Warburg effect (9, 13, 25). In lipid metabolism, it promotes *de novo* fatty acid synthesis by activating FASN through SUMOylation, contributing to tumor vascularization (28, 41). Within amino acid networks, PKM2 enhances serine biosynthesis by assembling enzyme complexes, supporting anabolic demands during rapid tumor growth (12, 47). Beyond metabolic regulation, PKM2 also establishes immunosuppressive microenvironments via STAT3 phosphorylation and HDAC3 recruitment, and mediates resistance to chemotherapy (4). These multifaceted roles identify PKM2 as a promising therapeutic target, particularly in tumors characterized by high metabolic plasticity. For instance, EZH2- and PKM2-mediated co-silencing of SLC16A9 in triple-negative breast cancer (TNBC) induces a metabolic shift from glycolysis to fatty acid oxidation, highlighting potential for synthetic lethality via dual-targeting strategies (114).

Although PKM2 is widely recognized for its oncogenic functions in digestive system malignancies, recent findings in other tumor types suggest that PKM2 may exhibit context-dependent tumor-suppressive roles. Notably, in head and neck squamous cell carcinoma, PKM2 has been reported to exert tumor-inhibitory effects, highlighting a bidirectional regulatory capacity (115, 116). This contrasts with the predominantly pro-tumorigenic role described in hepatocellular carcinoma, gastric cancer, colorectal cancer, and pancreatic cancer within the present review. Nevertheless, no direct experimental evidence within current digestive system tumor studies confirms comparable tumor-suppressive functions. Some observations imply inhibitory roles under particular modifications, but these findings are isolated and lack comprehensive mechanistic validation. For example, in HCC, ULK1-mediated phosphorylation at Ser333 enhances PKM2 enzymatic activity, limits its nuclear localization, and suppresses c-Myc expression, collectively attenuating the Warburg effect (61). In contrast, substantial evidence supports PKM2-mediated metabolic reprogramming, immune evasion, and drug resistance through STAT3 activation, lactate accumulation, and PD-L1 upregulation in gastrointestinal malignancies (4, 8, 13, 14). This suggests a predominantly pro-tumorigenic role in these tissues. The absence of confirmed suppressive functions may reflect tissue-specific regulatory inputs, differences in upstream signaling, or distinct metabolic dependencies. Future research should investigate whether PKM2 exhibits functional plasticity in digestive cancers through isoform-specific regulation, nutrient stress responses, and interactions with immune checkpoints or tumor suppressors (61, 110, 116).

Despite its multifaceted regulatory potential in metabolic reprogramming, current PKM2 research still faces limitations. Most mechanistic studies focus on PKM2’s role as an enzyme or kinase, while its function as a protein interaction hub involved in epigenetic regulation and RNA splicing remains underexplored, restricting an integrated understanding of its role in metabolic phenotype transitions. Clinically, PKM2’s dual regulatory properties may suppress T-cell function when inhibited, potentially leading to immune tolerance; thus, the therapeutic window and dosing control need further clarification (110). Additionally, dynamic nutrient fluctuations within the tumor microenvironment may trigger compensatory metabolic responses. For example, under low-glucose conditions, PKM2 inhibition paradoxically promotes cell survival, indicating that PKM2-targeted strategies must be evaluated in the context of microenvironmental status (111).

In response to these challenges, this paper systematically outlines the PKM2-mediated networks of glycolysis, lipid synthesis, and amino acid metabolism, summarizing its tissue-specific regulatory mechanisms across various digestive system tumors. These insights lay the groundwork for precision-targeted therapies and call for future research into PKM2’s context-specific roles, epigenetic functions, and immunological consequences.

Building on this foundation, a key area that warrants further investigation is the development of isoform-specific therapeutic strategies. Current inhibitors rarely distinguish between PKM2 and PKM1, risking disruption of physiological pyruvate flux and energy homeostasis in normal cells (4, 89). Given that PKM2-specific functions are largely dictated by alternative splicing and post-translational modifications, future research should prioritize the design of agents that modulate splicing regulators such as hnRNPA1 or lncRNAs including HOXB-AS3 to selectively suppress PKM2 isoform expression without impairing PKM1 activity (38, 69). Meanwhile, the dynamic interconversion between PKM2 tetramers and dimers offers an additional therapeutic axis that remains underutilized in drug development.

In parallel, the immunological implications of PKM2-targeted therapies demand careful re-evaluation. PKM2 not only drives tumor-intrinsic immune evasion by enhancing PD-L1 transcription and facilitating lactate accumulation but also regulates the metabolic programming of T cells, especially CD8^+^ TCF1^+^ subsets, through the pentose phosphate pathway (4, 8, 110). These dual and potentially opposing roles complicate the integration of PKM2 inhibition into immunotherapy regimens. Whether combinatorial strategies involving immune checkpoint blockade and PKM2 suppression would synergize or antagonize in gastrointestinal malignancies is currently unknown. Furthermore, how PKM2 impacts myeloid-derived suppressor cells, tumor-associated macrophages, or regulatory T cells within the digestive tumor microenvironment remains insufficiently characterized and warrants systematic exploration.

Beyond its metabolic and immunoregulatory capacities, PKM2 may also serve as a scaffold for chromatin and RNA regulatory complexes, functioning in ways that transcend enzymatic activity. Its reported interactions with HDAC3, PRMT6, and circular RNAs such as circMAT2B suggest that PKM2 plays a role in shaping epigenetic landscapes and transcriptomic plasticity under oncogenic stress (7, 19, 53). However, the spatial-temporal dynamics of these interactions, and their integration with metabolic cues such as nutrient depletion, hypoxia, or ROS accumulation, are not well defined. Future investigations into PKM2’s role in chromatin accessibility, alternative splicing, and long-range transcriptional regulation may yield novel insights into its non-canonical oncogenic functions.

Finally, the metabolic heterogeneity of the tumor microenvironment introduces additional complexity into PKM2-directed strategies. In glucose-limited conditions, digestive system tumor cells may shift toward lipid oxidation or glutamine catabolism, diminishing the efficacy of glycolysis-targeting agents and, paradoxically, rendering PKM2 inhibition survival-promoting (43, 82). Such context-dependent adaptations highlight the need for real-time metabolic profiling and companion diagnostics to stratify responsive tumor subsets. PKM2-targeted PET tracers or metabolomic signatures reflecting Warburg activity may aid in predicting treatment efficacy and guiding dosing (113).

In conclusion, as a central hub in tumor metabolic reprogramming, future research on PKM2 should focus on isoform-specific regulation, epigenetic functions, and immune modulation mechanisms. Beyond its metabolic roles, PKM2 participates in protein interactions, chromatin remodeling, and RNA splicing, necessitating deeper investigation of its spatial-temporal dynamics to uncover noncanonical oncogenic mechanisms. Designing precise inhibitors targeting PKM2 isoforms and modulating alternative splicing factors and post-translational modifications will enhance therapeutic specificity while minimizing effects on normal metabolism. The metabolic heterogeneity of the tumor microenvironment drives adaptive energy pathway shifts under nutrient stress, highlighting the need to integrate metabolomics and companion diagnostics for dynamic monitoring and patient stratification toward personalized therapies. Additionally, the dual role of PKM2 in immune cell metabolism and its interplay with immune checkpoint regulation calls for combinational strategies that pair immunotherapy with PKM2 inhibition. Integrating metabolic, immune, and epigenetic regulatory axes will offer novel insights and accelerate clinical translation of PKM2-targeted therapies in digestive system cancers, opening new frontiers for anticancer treatment.
